# Chicago Medical Society

**Published:** 1886-01

**Authors:** 


### SOCIETY REPORTS.

CHICAGO MEDICAL SOCIETY.

*Stated meeting December, 1885.* The President, C. T. PARKES, M. D., in the chair.

A CASE OF EXPULSION OF A LARGE SUB-MUCOUS FIBROUS TUMOR OF THE UTERUS, PER VIAS NATURALES, OR COLPO-MYOMOTOMY, was the title of a paper read by Professor J. H. ETHERIDGE. Mrs. A. B., aged 43 years, married 28 years, mother of three children, suffered from monorrhagia for six years past. She has had frightful hæmorrhages, but otherwise she has been healthy. In 1882, the first examination he was permitted to make disclosed a large sub-mucous, fibrous, uterine tumor, at the top of the fundus uteri, extending one inch above the umbilicus, its lateral diameter equaling its longitudinal diameter. A uterine sound was introduced eleven inches. There was no rectal or vesical disturbance. The only inconveniences experienced were the hæmorrhages and a "high stomach." The hæmorrhages have repeatedly brought her to death's door, but her recuperative powers were astonishing. Professor Etheridge then detailed her history for two years, during which time she had had several hæmorrhages, which were controlled by sponge-tents, rest and ergot. He detailed also the treatment by a homœpathic physician, which had consisted of incisions into the cervix uteri, and the administration of sulphuric acid and fluid extract of hydrastis canadensis, the latter remedy being extolled as a sure cure for uterine fibroids. On November 12, 1884, Professor Etheridge was called to see the patient, whom he found in a pitiable condition. She was exsanguinated, feverish and dyspeptic. She was placed on tonic treatment and rapidly convalesced. In October, 1885, she had another hæmorrhage, which lasted twenty-four hours, before a sponge tent was introduced. This tent was speedily forced out. Three more sponge-tents were subsequently introduced, and an enema of ninety minims of fluid extract of ergot was given. Powerful uterine contractions ensued; the capsule of the tumor was ruptured, and the hæmorrhage ceased. Three days after that one pound of the tumor was pulled and cut away. The tumor was pultaceous, friable and accompanied by a disgusting odor. On the day following, one-half of a pound of the tumor was taken away, and two days later as much more. Three days later the patient was put under the influence of ether, and Professor Etheridge attempted to remove the remainder of the tumor, bnt only succeeded in removing two pounds more. After this operation, the patient went into collapse and nearly died. However, she recuperated so rapidly that in five days thereafter he removed another pound of the tumor. Five days later he succeeded in getting away another pound and the pedicle. The uterus, during the ten days past, had decreased rapidly in size, and the fetid vaginal discharge had lessened. During the three weeks in which the six pounds of gangrenous mass were removed, he expected the patient would develop sepsis, but none occurred. As the patient was continually under the influence of ergot, and shreds of the tumor came away between the times of the operations, it is safe to estimate the weight of the tumor at thirteen pounds. The points of interest in the case were that the hæmorrhages recurred most severly in the autumn, which was probably due o the changes in the circulation incident to the occurrence of cold weather; and also that, in order to remove these tumors expeditiously, it is imperative that we should be enabled to draw the tumor down into the vagina, where it can be grasped, and, at the same time, to continuously contract the uterus behind the descending tumor so as to facilitate traction and to avoid hæmorrhage.

THE PRESIDENT remarked that this case teaches the lesson of the importance of early interference in fibrous tumors of the uterus, especially those that are large and accompanied by hæmorrhages, which facts indicate that they are close to the mucous membrane, and under the effect of the contraction of the muscular fibres of the uterus, and thus susceptible to the influence of ergot.

DR. JACOB FRANKS then detailed A CASE OF VESICAL CALCULUS, in which lithotrity was attempted. A Bigelow lithotrite was introduced, which grasped a stone measuring two inches. Upon turning the screw the instrument broke, and in consequence of the accident a portion of the instrument was left in the bladder. On the next day the lateral operation of lithotomy was performed, and the calculus and the broken piece of the instrument removed. The patient made a good covery.

The Sociey then adjourned.

---

CHICAGO MEDICAL SOCIETY.

*Stated Meeting, December 7th, 1885.* THE PRESIDENT, C. T. PARKES, M. D., in the Chair.

*Pneumonic Abscess* was the title of a paper by Dr. Edward F. Wells, of Minster, Ohio. He incorporated in his paper the histories of a number of cases which recovered under expectant and medical treatment. In an experience with 413 cases of pneumonia, abscesses occurred nine times. In closing his paper, the author said that he had arrived at the following conclusions :

1. The issue of pneumonic fever in abscess is rare, but this rarity has been greatly overestimated.

2. These abscesses vary much in size and are most frequently found at the base of the lung.

3. They may in some instances, after pneumonia, be caused by excessive jarring or other motion.

4. They are usually formed rapidly.

5. In some rare instances the purulent contents may degenerate into a cheesy mass, to again soften, liquefy and be discharged.

6. The symptoms and signs are quite distinctive and sufficient for an accurate diagnosis.

7. The majority of cases recover, but in a certain proportion a cure is impossible.

8. Expectant and medical treatment, thus far, has given the best results, and the majority of cases should be managed upon this plan, but, under certain conditions, the most radical measures for relief are not only justifiable but imperatively demanded.

PROFESSOR CHRISTIAN FENGER said that an interesting point in the paper is that the author takes the ground of the clinical and, perhaps, conservative man. Dr. Wells claims that we should not operate on the small abscesses, but only on large ones, as the latter are the more dangerous. Professor Fenger believed that we should operate on all of them when indicated. Buhl, in Christiania, has operated on nineteen cases in which there were cavities. Up to the present time, we have reports of about thirty cases in which operations have been performed, but only seven of these cases have been such as reported by

Dr. Wells, or abscesses incident to pneumonia. Since there have been only seven cases of abscesses of the lungs, incident to pneumonia, it stands to reason that these cases were severe ones, and that the abscesses were large. Professor Fenger did not believe there had been much operating on small abscesses as yet; however, he would advocate it in case these abscesses were accompanied by a fetid bronchitis. There is an abscess-cavity, which Rokitansky calls a chronic abscess, in which there is not connective tissue enough to allow the cavity to close. The cavity is a source of danger to the patient, as sometimes, for one reason or orther, septic micrococci gain entrance, acute inflammation or gangrene follows, and the patient dies. In such a condition, we should try to obliterate the cavity. Operative treatment of these abscesses has been so infrequent that we cannot say that it possesses any advantage over medical treatment. The important point to be decided in the future is how to get an understanding of where the danger-line is; how long we can afford to wait and how much strength can we afford to let the patient lose before we operate? It is, perhaps, well to put in a word of warning against operating too early, as we are all aware that patients who recover without an operation do better than those on whom there have been operations. Professor Fenger said that, in case a cavity gives rise to fetid breath, frequently a fetid bronchitis is developed, and subsequently an inter-lobular pneumonia in the other lung, or in the upper part of the lung in which the abscess exists. Relative to Dr. Well's remark that he never suppresses the cough, Professor Fenger stated that it is a fact that the cough ceases almost instantly after the abscess is opened.

DR. R. TILLEY said that he could not see of what benefit inhalations of turpentine could be in such cases, as the abscesses are analogous to those which occur in the most external portions of the body, and we never expect to benefit them by using turpentine. He would expect better results from administering this medicine internally, as he had, recently, read that the German physicians are using turpentine very successfully, by giving it internally, in diphtheria.

DR. WELLS, in closing the discussion, said that he wished to recall the attention of those present to the important point that these abscesses occur most frequently in the lower lobe of the lung, similarly to pneumonia. The principal object of his paper was to do what he could to counteract the tendency among the medical journals to advise early operation in pulmonary abscesses. He thought the statistics, so far, do not prove the operative treatment to be superior to the expectant and medical treatment, and he thought surgeons were too prone to take the cases into their own hands, and that they do not leave enough to the *vis medicatrix naturæ*.

